# 

**Published:** 1870-04

**Authors:** 


### Answers to Correspondents.

Mrs. L.—*Binghamnton, N. Y.*—We can only furnish the back numbers of Vol. V.

Dr. H.—*Athens, Pa.*—Geo. Tiemann & Co., whose address you will find in the Bistoury, will supply you.

D. W.—*Huntingdon, Pa.*—Cannot tell whether the disease mentioned is curable or not, without seeing the case. You will find our opinion of the Doctor (?) mentioned, under the head of Self-Styled Doctors.

I. M. B.—*Boonville, N. Y.*—The American Journal of Pharmacy can be had by addressing "Prof. Proctor, Editor of American Jour. Pharmacy," Philadelphia.

W. D.—*Parkville, Mo.*—You will find a recipe for preventing falling out of the hair, in No. 2 of Vol. V, page 50, of the Bistoury.

Mrs. T.—*Dunkirk, N. Y.*—"Have we ever had such a case?" Several. Let it alone, it will reveal itself in the course of nine months. This is the only prescription we have to offer you.

Dr. B.—*Van Ettenville, N. Y.*—We have written you, answering your queries. We are tired of publishing the names of Medical Book sellers through our columns without charge. See the point?

P. L. K.—*Jamestown, N. Y.*—Would we advise you to take any more of "Consumption Cure?" Of course we would. Also try "Phebe Baker's Salve," "Costar's Rat Exterminator," and "The English Sweeny Liniment." If that doesn't cure you, hang yourself, for you are beyond the reach of medicine.

Dr. B. F. C.—*Midway, Ala.*—You neglected stating what form of *herpes* your wife has. We will venture to suggest, for the part implicated, (the foot) that you bind about it a cloth, saturated with a strong solution of Sulphate of Copper.—℞. Cupri Sulphas. dr. i; Aquæ, f dr.vj. M. Continue this for a couple of weeks, gradually diluting the solution, daily. The parts should be kept continually wet with it.

LEFT OVER.—In consequence of the great length of Dr. Babcock's article on uterine difficulties, our Domestic Medicine Department has been crowded out. We will give you a double dose next time.

